# Supplementary material for: Effect of the Cu2+/1+ Redox Potential of Non-Macrocyclic Cu Complexes on Electrochemical CO2 Reduction
Source: Molecules. 2023 Jul 3;28(13):5179. doi: 10.3390/molecules28135179 (PMC10343594; doi:10.3390/molecules28135179)
Supplement: Supplementary file 1 [file molecules-28-05179-s001.zip › molecules-2469606-supplementary.pdf]

Effect of the $Cu^{2+/1+}$ Redox Potential of Non-Macrocyclic Cu Complexes on Electrochemical CO_2 Reduction

*Kyuman Kim, Pawel Wagner, Klaudia Wagner, and Attila J. Mozer**

Intelligent Polymer Research Institute and ARC Centre of Excellence for Electromaterials Science, University of Wollongong, Wollongong, New South Wales 2522, Australia

*Corresponding author.

E-mail: attila@uow.edu.au

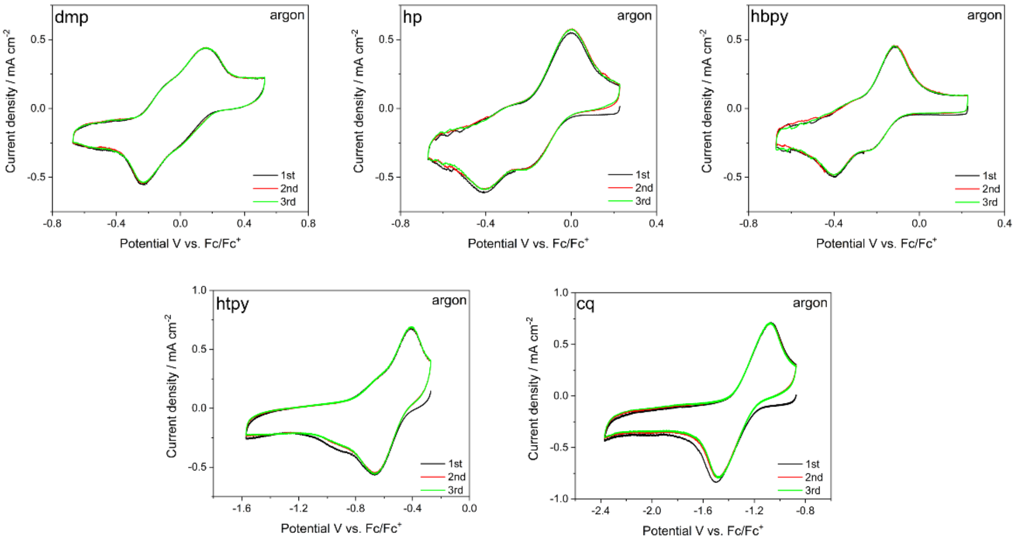

Figure S1. Three cycles of CV curves of five Cu complexes in argon

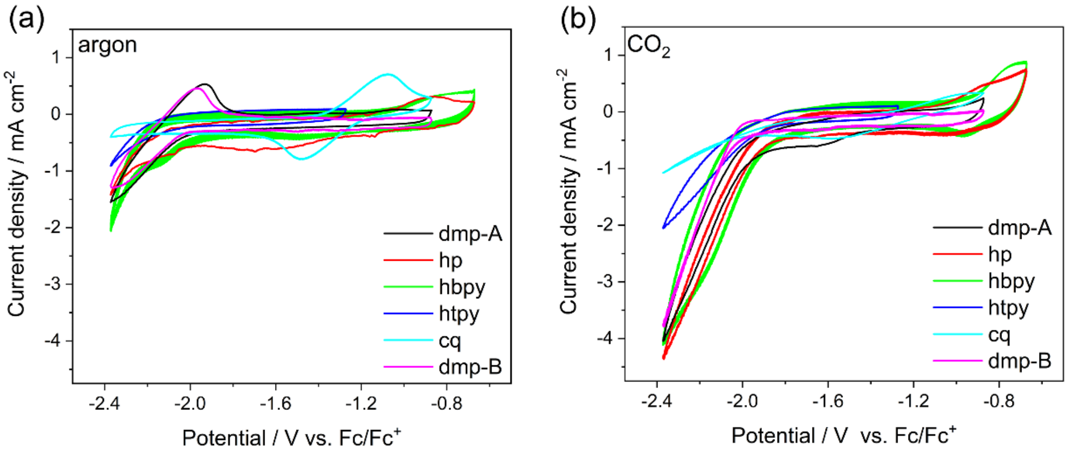

Figure S2. CVs of Cu complexes dissolved in 0.1 M TBAP/DMF electrolyte with 0.1 M water in argon (a) and CO_2 (b)

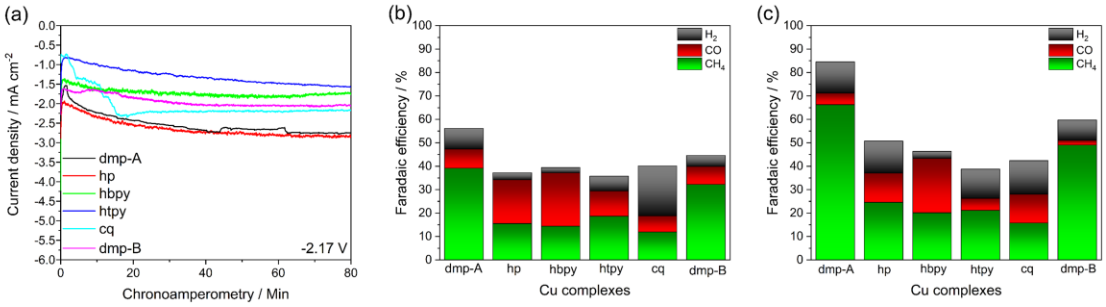

Figure S3. (a) CA at -2.17 V vs. Fc/Fc$^+$, (b) the FE of gas products at 20 min of CA and (c) FE of gas products at 80 min of CA

Electrode	Electrolyte	Atmosphere	H_2 (ppm) at -2.17 V
**Carbon paper**	**0.1 M TBAP/DMF + 0.1 M water**	**CO_2**	**10.9**
**Carbon paper**	**0.1 M TBAP/DMF + 0.1 M water + 1 mM cq complex**	**CO_2**	**342**

Table S1. The amount of H_2 produced using carbon paper with and without *cq* complex in the electrolytes during CA at -2.17 V vs. Fc/Fc^+

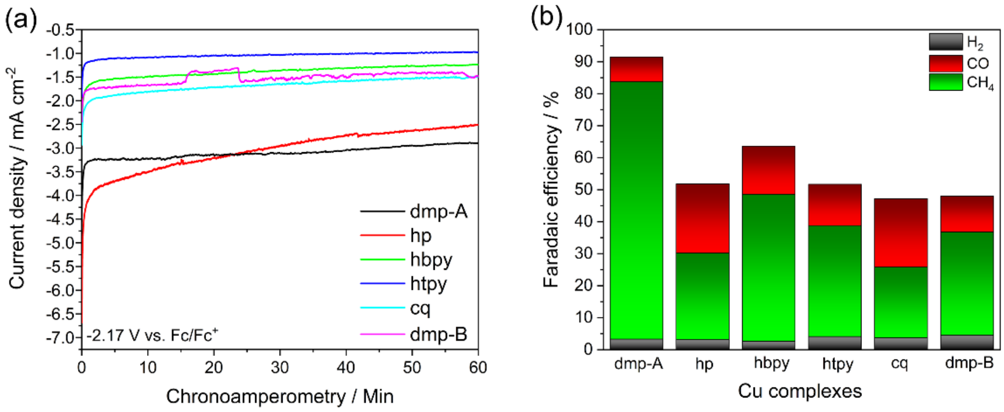

Figure S4. (a) CA in purely heterogeneous mechanism at -2.17 V vs. Fc/Fc^{+}, (b) FE of gas products at 60 min of CA

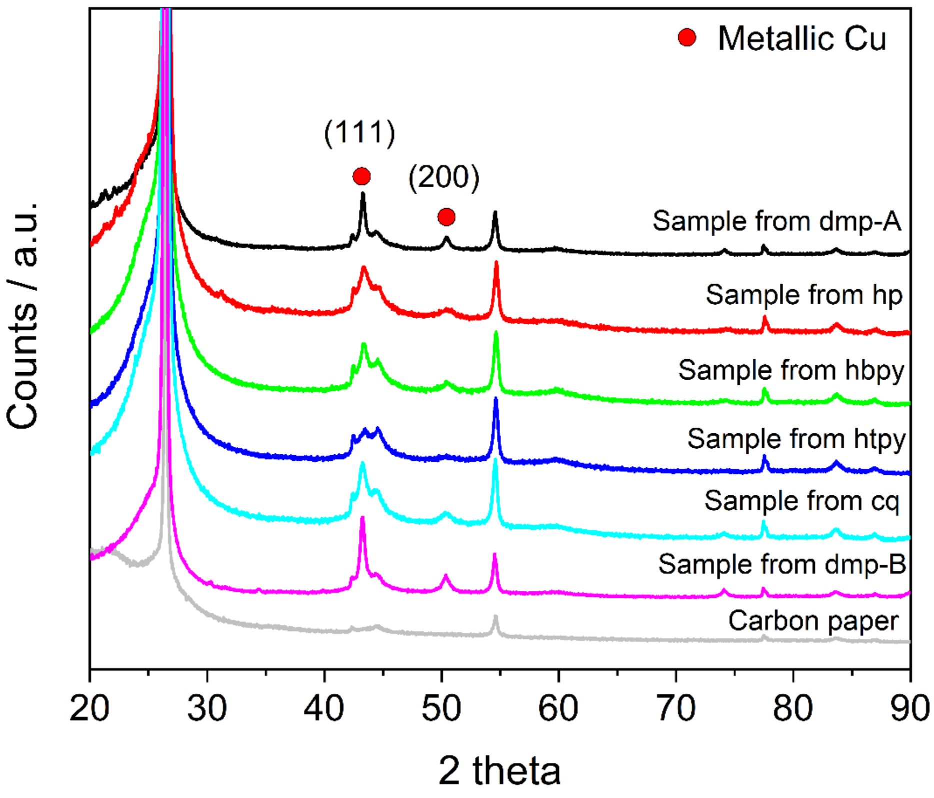

Figure S5. XRD patterns of carbon paper and Cu-complex-derived catalysts after CA.

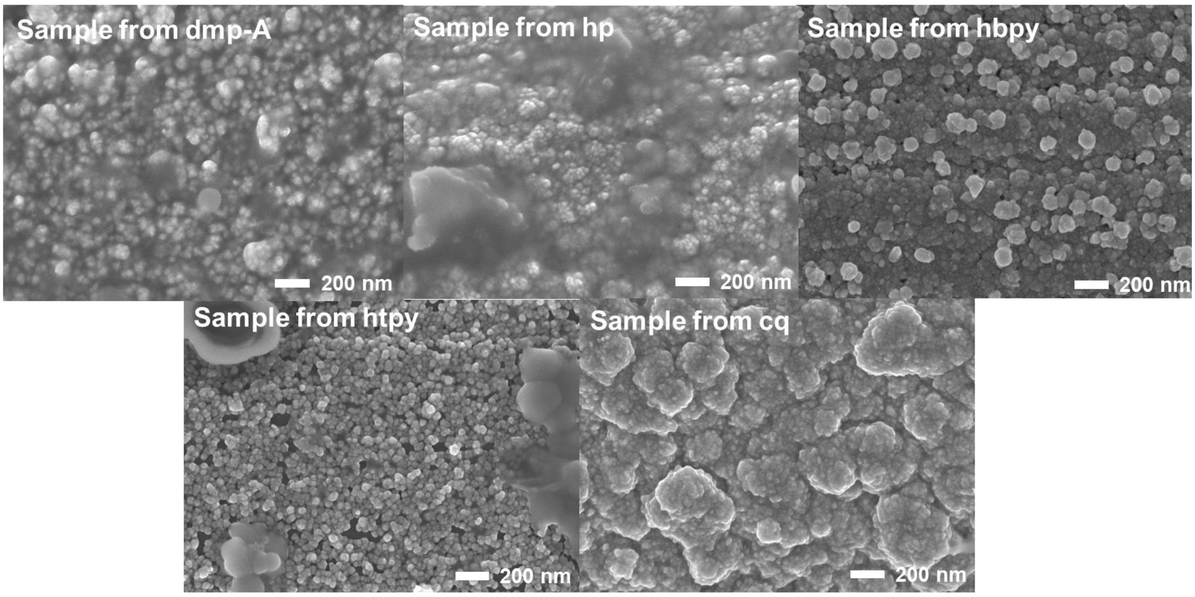

Figure S6. SEM images of Cu-complex-derived catalysts after CA

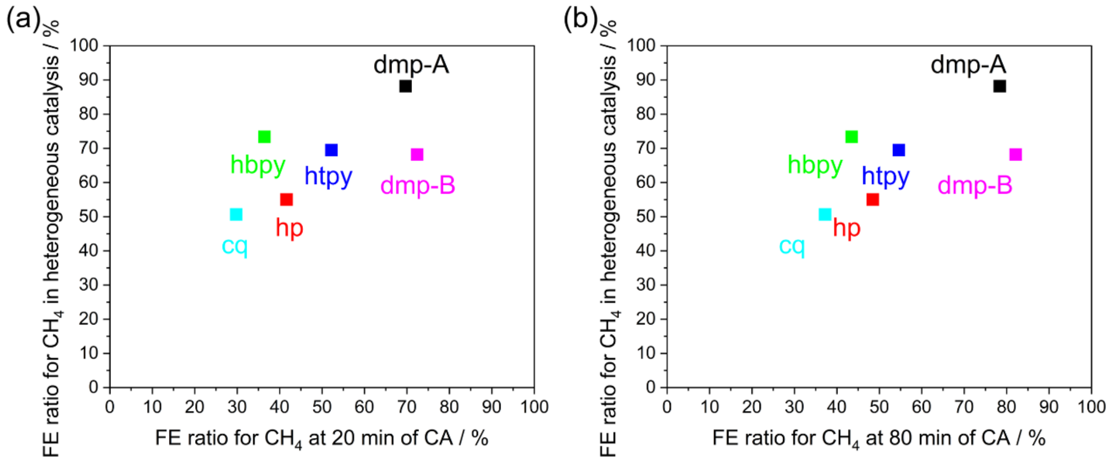

Figure S7. FE ratio for CH_4 in heterogeneous catalysis versus at 20 min (a) and at 80 min (b) of CA in the Cu complex dissolved electrolyte

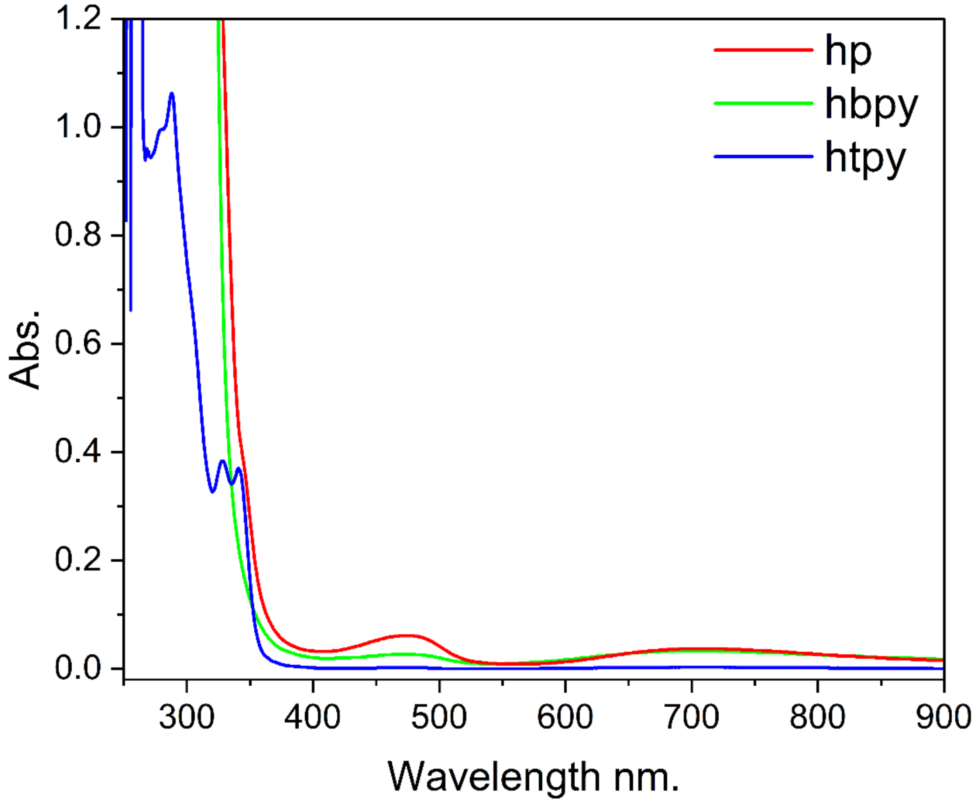

Figure S8. UV-vis spectra of Cu complexes synthesized using the HETPHEN strategy

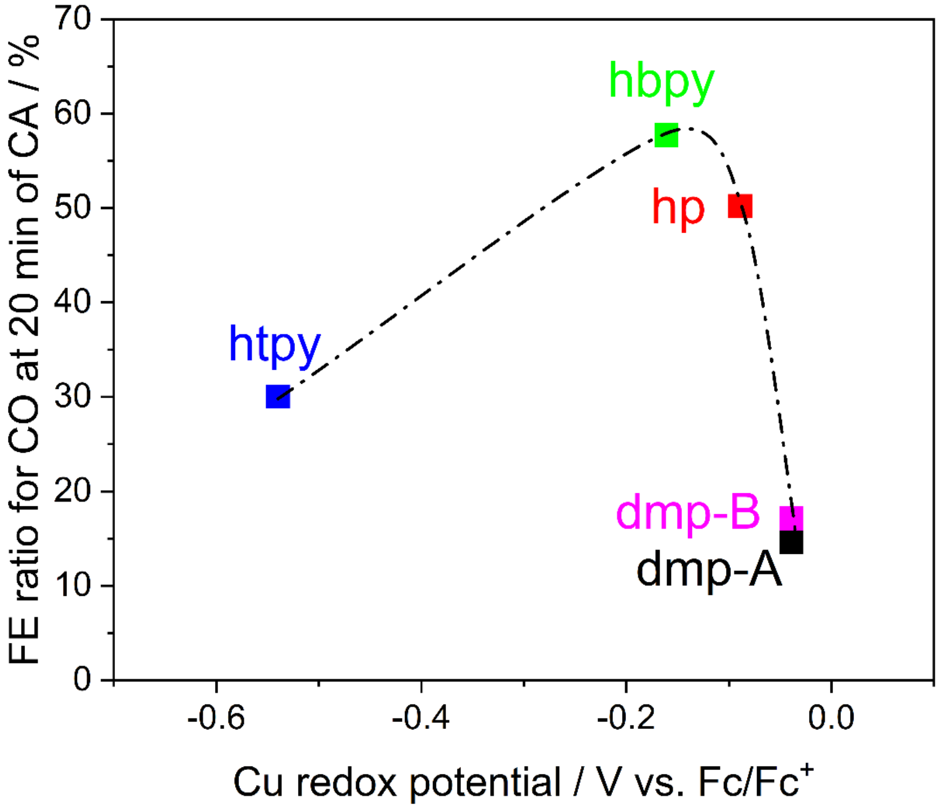

Figure S9. The Cu redox potential in argon (excluding *cq* complex) versus the Faradaic efficiency ratio of CO at 20 min of CA at -2.17 V vs. Fc/Fc^{+}

**Cu-complex-derived catalyst**	**Potential range (V vs. Fc/Fc$^+$)**	**Potential window (△V)**	**Specific capacitance (F/g)**
Sample from dmp-A	-1.2~-1.0	0.2	0.56
Sample from hp	-	-	-
Sample from hbpy	-1.6~-1.4	0.2	0.80
Sample from htpy	-1.6~-1.4	0.2	0.45
Sample from cq	-1.2~-1.0	0.2	0.65
Sample from dmp-B	-1.2~-1.0	0.2	0.43

Table S2. Specific capacitance of the Cu-complex-derived catalysts

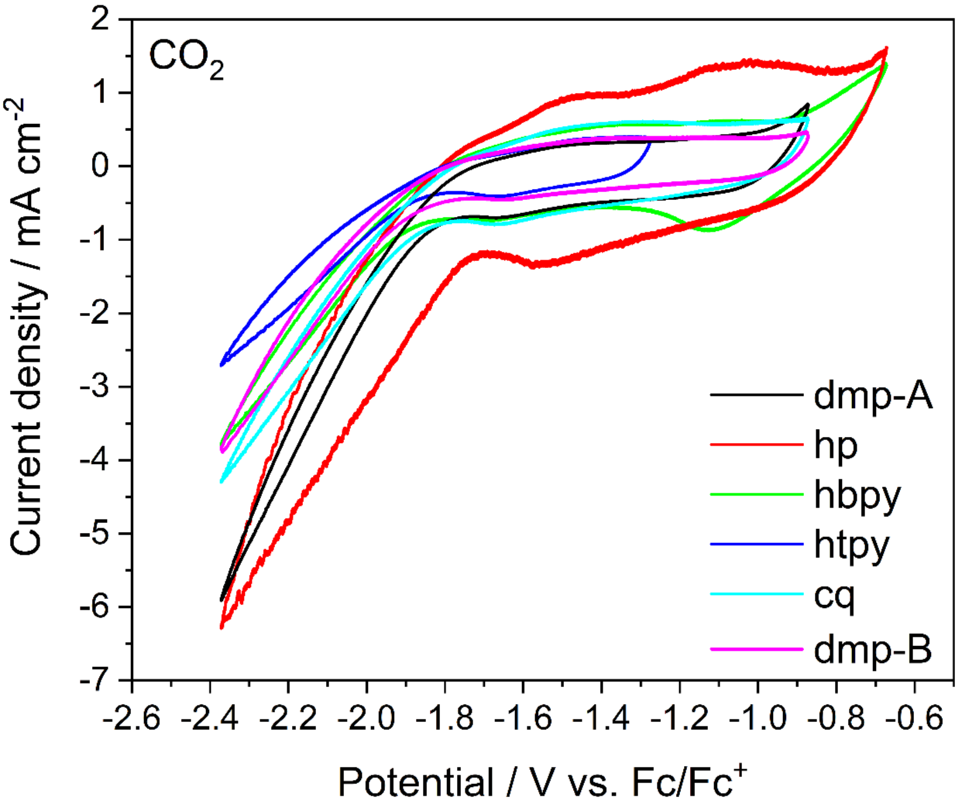

Figure S10. CVs of the Cu-complex-derived catalysts in CO_2

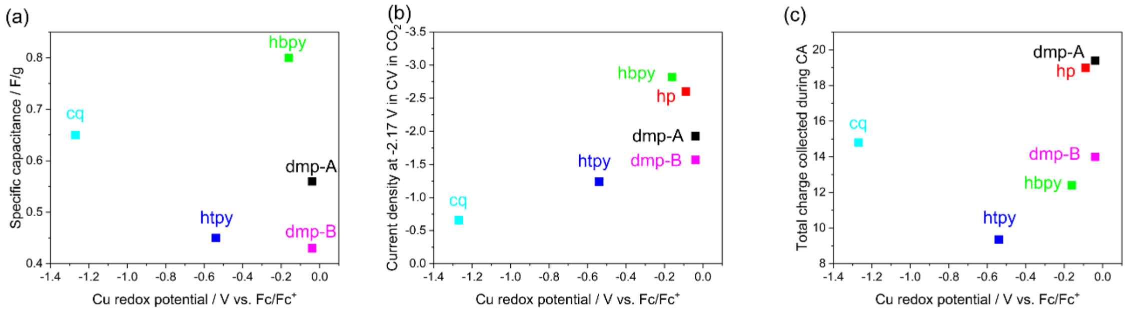

Figure S11. The Cu redox potential in argon versus specific capacitance (F/g) (a) and current density at -2.17 V in CV under CO_2 (b) and total charge collected during CA (c)
